# Differences in psycho-social impact of COVID-19 in albania, india and iran; a cross-section online study

**DOI:** 10.1192/j.eurpsy.2021.1754

**Published:** 2021-08-13

**Authors:** E. Dashi, R. Ransing, B. Vahdani, V. Alikaj

**Affiliations:** 1 Department Of Neuroscience, University Hospital Center “Mother Theresa”, Tirane, Albania; 2 Department Of Psychiatry, BKL Walawalker Rural Medical College, Ratnagiri-415606, Maharashtra, India; 3 Clinical Research Development Unit, Qazvine University Of Medical Sciences, Ministry of Health, Tehran, Iran; 4 Faculty Of Medicine, Tirana Medical University Albania, Tirane, Albania

**Keywords:** psycho-social impact, fear of covid, COVID-19, anxiety symptoms

## Abstract

**Introduction:**

After the outbreak of a new coronavirus infection (COVID-19) on 31 December 2019 in Wuhan (China), an increasing amount of information and concerns are impacting global mental health. It is already evident that apart from physical suffering, the direct and indirect psychological and social effects of COVID-19 pandemic are pervasive and could affect mental health now and in the future.

**Objectives:**

The central aim of our study was to investigate the prevalence of common mental disorders in populations during Covid-19 outbreak. The study was done in 3 different countries (Albania, India, Iran) which gave us the opportunity to compare our findings and to have a bigger view of the impact of COVID-19 in individuals.

**Methods:**

A Cross-sectional online survey was done across countries. We used demographic questions and different scales: Corona Anxiety Scale (CAS), The Obsession with COVID-19 Scale (OCS), Insomnia Severity Index (ISI) to evaluate psycho-social impact during covid.

**Results:**

We collected 469 responses in Albania, 442 responses in India and 402 responses in Iran. According to the data we found that symptoms of anxiety related with corona and obsession with corona were higher in Iran compared with the two other countries. Insomnia problems were also more frequent in Iran were only 16,66% of participants reported no insomnia, compared with 42,0% in Albania and 63,12% in India.

**Conclusions:**

The emergence of mental health (MH) problems during a pandemic is extremely common, though difficult to address due to the complexities of pandemics.

**Disclosure:**

No significant relationships.

